# Nephrotic-range proteinuria in a 53-year-old female with thin basement membrane nephropathy: A case report with literature review

**DOI:** 10.1177/2050313X251365367

**Published:** 2025-08-11

**Authors:** Ahmad Matarneh, Sundus Sardar, Pankhuri Mohan, Omar Salameh, Catherine Abendroth, Ronald Miller, Nasrollah Ghahramani, Navin Verma

**Affiliations:** 1 Department of Nephrology, Pennsylvania State Milton S. Hershey Medical Center, PA, USA; 2 Internal Medicine–Pediatrics Residency Program, Pennsylvania State Milton S. Hershey Medical Center, PA, USA; 3 Department of Nephrology, University of Missouri–Kansas City Medical Center, MO, USA; 4 Department of Pathology, Pennsylvania State Milton S. Hershey Medical Center, PA, USA

**Keywords:** electron microscopy in nephropathy, nephrotic syndrome, thin basement membrane nephropathy (TBMN), nephrotic-range proteinuria, proteinuria management

## Abstract

Thin basement membrane nephropathy is traditionally characterized by persistent microscopic hematuria and minimal proteinuria, and has long been considered a benign condition. However, emerging evidence challenges this view, as thin basement membrane nephropathy can occasionally present with nephrotic-range proteinuria. We report a case of a 53-year-old female with isolated nephrotic-range proteinuria, ultimately diagnosed with thin basement membrane nephropathy based on electron microscopy findings. Her proteinuria improved significantly over a 10-month period with angiotensin-converting enzyme inhibitor therapy, lisinopril 40 mg once daily. This case adds to the growing recognition that thin basement membrane nephropathy may not always follow a benign course and highlights the importance of careful evaluation and management when clinical features fall outside the expected pattern. A comprehensive literature review is also provided, summarizing similar presentations and management strategies.

## Introduction

Thin basement membrane nephropathy (TBMN), also known as benign familial hematuria, is one of the most common inherited glomerular diseases, affecting ~1% of the population. However, epidemiological data on atypical presentations—such as isolated proteinuria without hematuria—are scarce, with only sporadic case reports available in the literature. It is primarily caused by heterozygous mutations in the *COL4A3* or *COL4A4* genes, which encode the α3 and α4 chains of type IV collagen, essential components of the glomerular basement membrane (GBM).^1^ These genetic findings not only aid in diagnosis but also underscore the importance of familial screening to identify at-risk relatives. Furthermore, they have implications for patient counseling, prognostication, and early intervention. Clinically, TBMN typically presents with persistent microscopic hematuria, minimal proteinuria, and preserved renal function, and is historically considered a benign condition with an excellent prognosis.^2^

However, recent case reports have challenged this assumption by describing TBMN presentations with nephrotic-range proteinuria and, in some instances, progression to chronic kidney disease (CKD).^3^ This report describes a 53-year-old woman who presented with isolated nephrotic-range proteinuria in the absence of hematuria, later diagnosed as TBMN based on electron microscopy (EM) findings. Genetic testing was not performed due to cost and limited availability, a common barrier in clinical practice that highlights the ongoing gap between genomic insights and real-world implementation in atypical TBMN cases. This case highlights how careful histopathologic evaluation can still play a crucial role in reaching a confident diagnosis. A comprehensive literature review is provided to explore the clinical variability of TBMN and to review diagnostic challenges and therapeutic approaches in such atypical cases.

## Case presentation

A 53-year-old woman with a history of hypertension, hyperlipidemia, and hypothyroidism, each medically managed, was referred to nephrology for evaluation of newly identified proteinuria. She had no prior kidney disease, and her renal function had been normal on routine labs.

She reported several weeks of intermittent lower extremity edema but denied urinary symptoms, hematuria, or systemic complaints such as fever, fatigue, weight loss, or rash. There was no family history of kidney disease, hematuria, or autoimmune conditions.

The proteinuria was first noted during a routine urinalysis at a cardiology visit for dyslipidemia management, which revealed 4+ protein without hematuria. A spot urine protein-to-creatinine ratio confirmed nephrotic-range proteinuria (4.19 g/g). Serum creatinine was normal with an estimated glomerular filtration rate (eGFR) >60 ml/min/1.73 m^2^. Serum albumin was mildly reduced at 3.3 g/dl, and lipid levels were elevated. Repeat urinalysis confirmed persistent proteinuria without microscopic hematuria.

Further workup included a positive a﻿nti n﻿uclear antibodies (ANA) at 1:160 with a homogeneous pattern, but follow-up testing, including anti-dsDNA, ENA panel (Sm, RNP, SSA/Ro, SSB/La, Scl-70, Jo-1), ANCA, and anti-GBM antibodies were negative. Complement levels were normal. Tests for hepatitis B, C, and HIV were negative. There was no evidence of monoclonal gammopathy on serum and urine electrophoresis or free light chain assay. Thyroid function was normal, and anti-thyroid antibodies were absent.

Given the isolated nephrotic-range proteinuria, preserved renal function, and unrevealing serologic workup, minimal change disease (MCD) was initially suspected. We performed a native kidney biopsy to establish a definitive diagnosis.

## Histopathological findings

**Figure 1.**
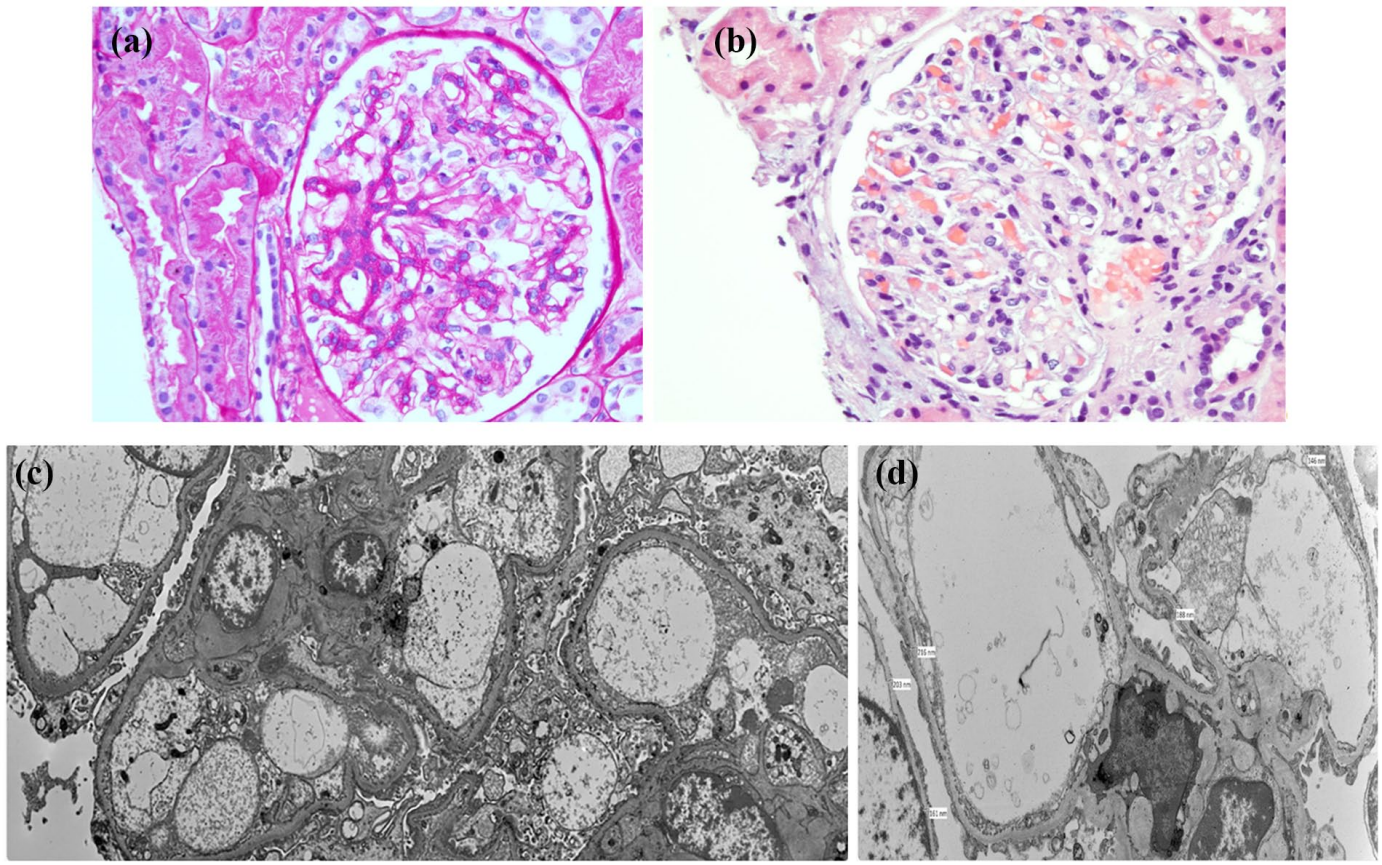
(a) Periodic acid–Schiff stain shows a glomerulus with normal morphology, lacking mesangial expansion or sclerosis. (b) Hematoxylin and eosin stain confirms preserved glomerular architecture with no evidence of endocapillary hypercellularity or necrosis. (c) Electron microscopy reveals segmental podocyte foot process effacement (<50% of capillary loop surface), insufficient to support a diagnosis of minimal change disease. (d) Uniformly thinned glomerular basement membrane measuring 161–216 nm (normal ~330 nm), consistent with TBMN. No immune deposits or structural abnormalities were identified. TBMN: thin basement membrane nephropathy.

Representative light microscopy and EM findings are shown in Figure 1.

### Light microscopy

Two renal cortical cores containing 30 glomeruli were examined. The glomeruli appeared normal in size and cellularity, with no mesangial expansion, endocapillary hypercellularity, segmental sclerosis, necrosis, or crescents. Silver staining showed an intact basement membrane without evidence of spikes or duplications. The interstitium showed mild fibrosis and tubular atrophy, along with a minimal lymphocytic infiltrate. These findings were consistent with a non-proliferative, non-inflammatory glomerular process. In the absence of immune complex deposition and with preserved architecture, MCD was initially considered.

### Electron microscopy

EM revealed diffusely and uniformly thinned GBMs, measuring between 161 and 216 nm. For comparison, normal adult GBM thickness is generally around 330–350 nm. Foot process effacement was present but limited to <50% of the capillary surface area—well below the threshold typically seen in MCD, where diffuse effacement of at least 80% is expected. No electron-dense deposits or structural abnormalities, such as GBM splitting or lamellation, were seen. These findings confirmed a diagnosis of TBMN and excluded MCD and other immune-mediated glomerular diseases. Immunohistochemical staining for type IV collagen α5 was not performed, but could have helped differentiate TBMN from early Alport syndrome.

## Diagnosis and clinical course

Based on the combination of clinical features, unremarkable systemic workup, and kidney biopsy findings, we diagnosed the patient with TBMN presenting with isolated nephrotic-range proteinuria. Importantly, there was no hematuria, immune complex deposition, or additional glomerular pathology identified.

Given the non-inflammatory nature of TBMN and the absence of a coexisting glomerulopathy such as focal segmental glomerulosclerosis (FSGS) or MCD, immunosuppressive therapy was not indicated. Instead, we started the patient on an angiotensin-converting enzyme inhibitor (ACEI) for renoprotection and to reduce proteinuria. Lifestyle and dietary counseling were provided, with a focus on salt restriction and lipid management. Table 1 shows the pertinent laboratory findings before and after r﻿enin angiotensin aldosterone system (RAAS) blockade. At the initial nephrology visit, her blood pressure was measured at 148/92 mmHg. Given the role of hypertension in worsening proteinuria and potentially accelerating TBMN progression, we started lisinopril at 40 mg once daily. Over the next month, her blood pressure improved to 132/84 mmHg, and by the 3-month follow-up, it stabilized at 128/80 mmHg. She tolerated the medication well, without hypotension or changes in kidney function, and the dose remained unchanged throughout follow-up.

**Table 1.** Serial laboratory values before and after RAAS blockade.

Lab value	Reference value	Initial evaluation	After RAAS blockade
Hb	12–14 g/dl	12.2	11.7
Creatinine	0.7–1.3 mg/dl	0.97	0.94
ANA	Negative	1:160	–
Albumin	3.5–4 g/dl	3.3	3.5
Urine WBC	0–4	0–4	0–4
Urine RBC	0–4	0–4	0–4
Urine protein	Negative	⩾500	+100
Urine protein/creatinine ratio	<0.15 g/g	4.19	2.49

At follow-up visits over the next several months, the patient remained asymptomatic and adherent to therapy. She reported resolution of lower extremity edema and experienced no adverse effects from the ACEI. Laboratory monitoring demonstrated progressive improvement in proteinuria, with a decline in spot urine protein to creatinine ratio (UPCR) from 4.19 to 2.49 g/g, and a modest increase in serum albumin from 3.3 to 3.5 g/dl. Serum creatinine remained stable, and eGFR continued to exceed 60 ml/min/1.73 m^2^.

**Figure 2.**
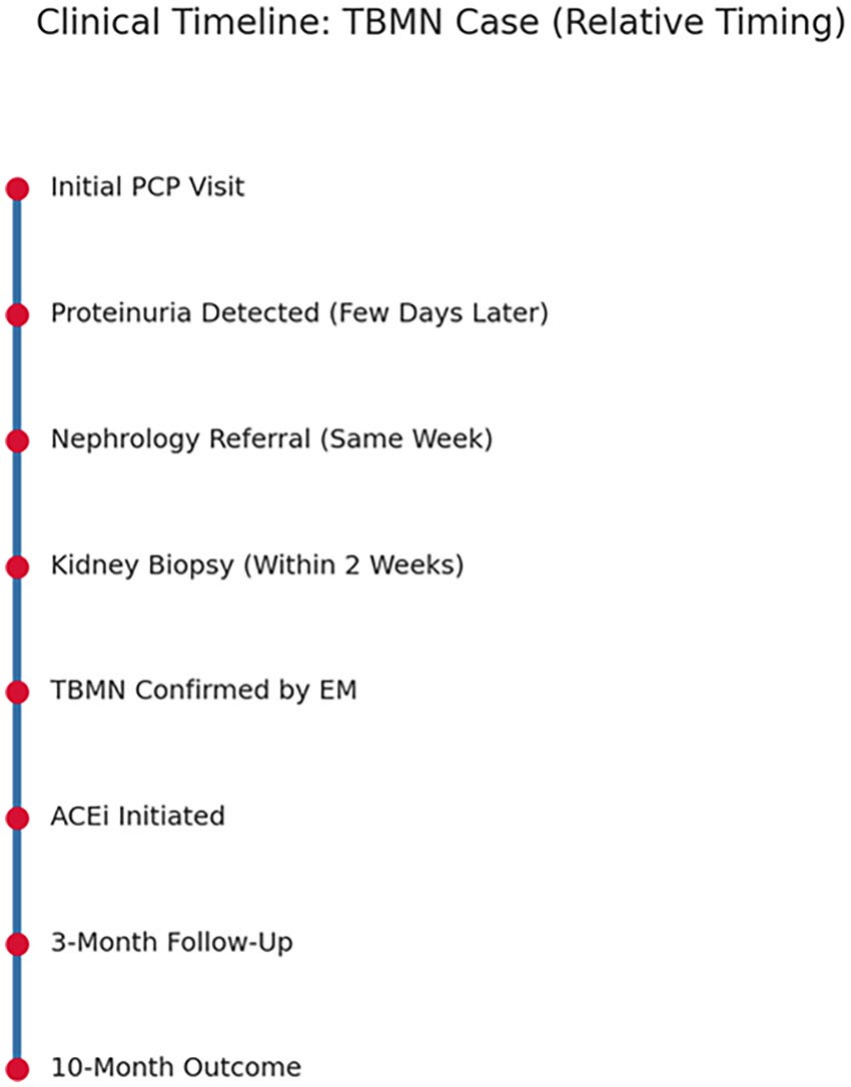
Approximate clinical timeline showing the key diagnostic and therapeutic milestones, including initial urinalysis (week 0), nephrology referral (week 2), kidney biopsy (week 5), and ACE inhibitor initiation (week 6) in a 53-year-old woman with isolated nephrotic-range proteinuria due to TBMN. ACE: angiotensin-converting enzyme; TBMN: thin basement membrane nephropathy.

She continued to be followed regularly in the nephrology clinic for monitoring of proteinuria, kidney function, and blood pressure. The absence of disease progression, normalization of serum albumin, and stable renal function all supported the benign course of TBMN in this patient, despite its atypical presentation with nephrotic-range proteinuria. The key diagnostic and therapeutic events in this case are summarized in the clinical timeline (Figure 2).

We continued to monitor her regularly in the nephrology clinic. Proteinuria declined, serum albumin normalized, renal function remained stable, and blood pressure was well controlled. These findings supported a favorable course consistent with the benign nature of TBMN, despite its atypical presentation. Although genetic testing was not performed, we discussed it as an option if her clinical picture evolved or family history became relevant. Figure 2 summarizes the diagnostic and therapeutic milestones.

## Discussion

TBMN is typically considered benign, but reports increasingly describe cases with nephrotic-range proteinuria and renal impairment.^4^ This discussion explores the pathophysiologic mechanisms, clinical implications, and management strategies relevant to TBMN presenting with significant proteinuria.

A leading hypothesis suggests that sustained protein leakage across a thinned GBM induces secondary podocyte injury, resulting in foot process effacement, a hallmark of nephrotic syndrome.^5^ Chronic proteinuria may initiate glomerular injury, eventually leading to FSGS. This process can worsen proteinuria and contribute to progressive renal dysfunction.^6^

Nephrotic-range proteinuria may signal progression, particularly in patients with hypertension or diabetes.^7^ Notably, the age of onset for nephrotic-range proteinuria in TBMN varies widely, from early childhood to late adulthood.^8^ This heterogeneity points to underlying genetic and clinical modifiers that likely influence TBMN outcomes.

A brief review of the literature identified several case reports of TBMN presenting with nephrotic-range proteinuria. The details of these cases, including patient demographics, biopsy findings, and outcomes, are summarized in Table 2.

**Table 2.** Reported cases of TBMN with nephrotic-range proteinuria.

Case	Patient details	Hematuria	Proteinuria	Findings	Treatment	Outcome	Key feature
Vaidya and Elsayed^2^	53-Year-old male	Yes	Nephrotic range	Isolated TBMN, no other glomerulopathies	ACEI	CKD Stage III	Similar age; hematuria present
Yoshida et al.^4^	Elderly Japanese woman	No	Nephrotic syndrome	Thin GBM, partial foot process fusion	ACEI	Remission	No hematuria; responded to ACEI
Ishimori et al.^3^	3-Year-old boy	Yes	Nephrotic range	Diffuse GBM thinning (~150 nm)	ACEI	Remission	Very young; excellent response
Fujinaga et al.^5^	15-Year-old boy	Yes	Nephrotic syndrome	TBMN + minimal change disease	Steroids	Remission	Dual pathology; required immunosuppression
Polito and Polito^14^	Adult woman	Not stated	Nephrotic syndrome	TBMN and IgA nephropathy	Not reported	Not reported	Double glomerulopathy
Deltas et al.^13^	Carriers of ARAS	Yes	Some with proteinuria	TBMN → FSGS later in life	Variable	Progressive CKD	Genetic overlap; FSGS progression
Current case (Matarneh et al. 2025)	53-Year-old female	No	Nephrotic range	Isolated TBMN confirmed by EM	ACEI (Lisinopril 40 mg daily)	Improved over 10 months	No hematuria; isolated TBMN

ACE: angiotensin-converting enzyme inhibitor; CKD: chronic kidney disease; EM: electron microscopy; FSGS: focal segmental glomerulosclerosis; TBMN: thin basement membrane nephropathy; ARAS: a﻿utosomal recessive a﻿lport syndrome.

When proteinuria is disproportionate, a precise diagnosis is essential to rule out coexisting glomerular disease. While EM remains the gold standard for identifying the characteristic thinning of the GBM, the presence of significant proteinuria should prompt a thorough evaluation to exclude other coexisting or mimicking glomerular diseases, such as MCD, membranous nephropathy, or FSGS.^9^ Given the potential for overlap, a kidney biopsy is often necessary to distinguish TBMN from these proteinuric conditions. In addition to histopathologic evaluation, genetic testing for COL4A3 and COL4A4 mutations can provide definitive confirmation of TBMN, particularly in patients with a family history of kidney disease or persistent proteinuria.^10^

Immunohistochemical analyses, including staining for type IV collagen α5, can further aid in differentiating TBMN from hereditary nephropathies such as Alport syndrome, which may present with similar clinical findings but carries a higher risk of progressive kidney disease.^11^ A thorough family history is essential, as TBMN often follows an autosomal dominant inheritance pattern and may be identified across multiple generations. Genetic mutations in TBMN often involve heterozygous variants in the COL4A3 and COL4A4 genes, which encode type IV collagen critical to the GBM. Patients usually inherit these mutations in an autosomal dominant fashion.^12^ In our case, we did not pursue genetic testing due to limited access and the patient’s stable clinical course. She reported no known family history of kidney disease. We discussed the potential genetic basis of TBMN, including the role of COL4A3 and COL4A4 mutations. Although we did not initiate formal family screening, we addressed the possibility during clinical discussions and noted that it could be reconsidered if new clinical concerns or family history emerged. Given the overlap between TBMN and autosomal dominant Alport syndrome, future genetic evaluation may help clarify prognosis and guide management if needed.

Management strategies should be individualized, but RAAS inhibition remains the cornerstone of therapy for TBMN with significant proteinuria, which reduces proteinuria and protects renal function. In cases where TBMN coexists with other glomerulopathies, such as MCD or FSGS, immunosuppressive therapy may be warranted.^13^ In these cases, corticosteroids or calcineurin inhibitors may be considered, particularly when proteinuria is severe or kidney function is declining.^14^ However, the decision to initiate immunosuppression must be made cautiously, balancing potential benefits with the risks of adverse effects, including metabolic complications, infection risk, and nephrotoxicity. It is important to emphasize that, unlike immune-mediated nephropathies, TBMN itself does not involve inflammatory processes, meaning that steroids and other immunosuppressive therapies have no direct role in treating TBMN in isolation.^15^

Prognosis varies depending on the severity of proteinuria, the presence of comorbidities, and underlying genetic mutations. Isolated TBMN generally has a favorable outlook, while patients with persistent proteinuria or genetic variants affecting COL4A3/COL4A4 may progress to CKD.^16^

Risk factors associated with worse outcomes include persistent proteinuria exceeding 500–1000 mg/day, hypertension, and a declining eGFR.^17^ For these patients, close monitoring is essential to detect early signs of disease progression. Regular assessment of serum creatinine, eGFR, proteinuria levels, and blood pressure is necessary to guide treatment adjustments and early intervention. Given that some patients with TBMN may experience worsening kidney function despite initial stability, timely recognition of progressive disease is critical to optimizing management and potentially altering the disease trajectory.

Patient Perspective: The patient expressed relief in receiving a clear diagnosis and was satisfied with the noninvasive management plan. She reported that the improvement in her symptoms significantly enhanced her quality of life. While genetic testing or family screening was not pursued, we briefly discussed the inherited nature of some glomerular diseases and the typically benign course of TBMN. She was reassured by the benign nature of TBMN and valued the plan for continued monitoring. We advised continued monitoring with quarterly assessments of serum creatinine, UPCR, and blood pressure. Re-biopsy may be considered if hematuria develops, proteinuria worsens, or eGFR declines.

## Conclusion

Our case reinforces that TBMN does not always follow a benign course. Even in the absence of hematuria or genetic confirmation, nephrotic-range proteinuria may indicate clinically significant disease. Early diagnosis and initiation of RAAS blockade may support renal stability and mitigate progression.
